# Sanatory State of Large Towns

**Published:** 1845-01

**Authors:** 


### FIRST REPORT OF THE COMMISSIONERS FOR INQUIRING INTO THE STATE OF LARGE TOWNS AND POPULOUS DISTRICTS. Vol. the First and Second. 1844.

[Second Article.]

A GLANCE over Good's Nosology, where we survey the melancholy catalogue of ills to which flesh is heir, is enough to appal the stoutest heart; but a perusal of the miseries, malarias, and morbific agencies, laid open by the SANATORY COMMISSIONERS, may well "frighten the isle from its propriety," and drive into madness and despair the human philanthropist! But the philanthropist is not always the philosopher. From a wide survey of human nature, he learns the mournful fact, that misery and death form a part of the wise system ordained by Nature's God for the government of mankind—and even of the brute creation. Were there no diseases and causes of diseases to curtail the natural range of existence, how long would the earth we inhabit afford food for the inhabitants? Even as it is, the population, in spite of bad drains, malaria, crowding, &c., with "the varying intensity of their operations," rapidly increases, rendering each successive generation more straightened for room, more worn by competition, more stinted in food, clothing, and wages, than their predecessors! Still, the *rate* of redundancy is greatly checked by these sad evils, that seem the inevitable lot of humanity. Suppose, for example, that by some strange combination of circumstances, the manufacturers in this country were, all at once, enabled to increase the wages of their workmen three-fold—to raise them from ten to thirty shillings a week? What would be the consequence? Marriages would be infinitely more numerous, and more early contracted—children would multiply more quickly—parents and children would live longer, from the increase of their comforts, both in food, clothing, and lodging—and, finally, the rate of increase in population would be doubled or trebled. In a few years—certainly in a few generations, the redundancy would be such, that wages would inevitably fall, from the excessive competition—and the miseries, privations, and premature decay, which we are now bewailing and endeavouring to remove or prevent, would accumulate to a similar or greater extent than they do at the present moment! Thus, the philanthropists are travelling in a circle. They are clearing away the *causes* of sickness and death, while their humane efforts are constantly tending to increase the original *fons et origo* of the evil—redundancy of population!

Nor can the philanthropist and the Christian halt in their humane endeavours to remedy ills that are, in fact, irremediable. The *immediate* causes and their dreadful *effects* are around them and before their eyes—the remote, but master-cause, is at a distance—dimly seen—little considered—and too generally denied. All the best feelings and impulses of our nature are enlisted in the attempt to *palliate* miseries which we cannot either cure or prevent. Charity, pity, hope, religion, morality, and humanity, all combine to urge us forward in the noble, though fruitless labour of expelling poverty, sickness, and death from the crowded cellars, hovels, mines, and factories of a redundant population!

In our last number we gave a general and comprehensive view of the Sanatory Report, and shall confine ourselves, in this article, to some particulars of a more local and specific character.

1. *Dr. Southwood Smith.*—The examination of this able and observant physician leads the van in these volumes. He states that the neglect of cleansing and drainage are the grand causes of sickness among the poor of the metropolis—especially in the "fever districts"—viz. "those localities where fever is *always* so prevalent as to be regarded as the ordinary seat of the disease."

"The fever districts of the metropolis are situated in different parts of it; and it is in accordance with ordinary experience to find fever raging in some of these districts, at the very time that others are enjoying a temporary immunity from it. In former years I have found, on my personal examination, some localities in which there was not a single house in which fever had not prevailed, and in some cases not a single room in a single house in which there had not been fever. I observed this particularly in certain localities in Bethnal-green and Whitechapel: now, during the present year, there has been a very remarkable absence of fever in these its ordinary seats, while in other districts it has been more than commonly prevalent." 2.

There is some discrepancy here. If a fever district *always* presents fever, how is it that one district will be free, and another scourged by the malady, at the same time—the bad drains and filth remaining the same? There must, therefore, be more than malaria engaged in the generation of fever, notwithstanding the "immediate neighbourhood of uncovered sewers, stagnant ditches and ponds, gutters always full of putrefying matter, nightman's yards, and privies the soil of which lies openly exposed, and is seldom or never removed."

It is not a little remarkable that Bethnal Green and Whitechapel should ever be free an hour from fever under such circumstances as above! But the essential cause of epidemic fever is just as little known as it was in the days of Hippocrates. That malaria, however, is a potent auxiliary to the invisible fiend, cannot be doubted, from the increased ratio of mortality in the malarious districts. It must be borne in mind, however, that wherever we find filth, crowding, and bad drainage, we also find poverty, with all its moral, physical, and mental miseries, ten thousand times more destructive to health than all the stinks that ever issued from the banks of Thames, Tyber, or Rhine.

Dr. Smith remarks, that the period of life most liable to fever, is that which intervenes between twenty and forty years—so that it (fever) may be considered as *the* disease of adolescence.

"Two consequences follow of the highest interest and importance. First, it is clear from these tables that the period of human existence during which fever can alone be said to be prevalent is from the age of twenty to forty; that is, the period of maturity, the most precious portion of the term of existence, that during which the individual is best fitted for all the duties and enjoyments of life, during which he is most capable of promoting the happiness of others, and of securing and appreciating his own. But of this period that portion which is incomparably the most subject to the ravages of this malady is the earliest portion. Now it must be borne in mind that the poorer classes usually marry and have families at earlier ages than the middle and higher, the great majority, at least of the women, being married at twenty. Of course it is during the succeeding ten years that they have young families, often very numerous ones, to support; but we have just seen that this is precisely the ten years in which fever is so prevalent as to furnish, in this comparatively short space of time, nearly as many cases as all the other periods of life put together. It follows, not only that the heads of families are more subject to the ravages of fever than any other class of persons, but that these persons are peculiarly liable to be attacked precisely at that period of life when they have the greatest number of young children entirely dependent on their daily labour for support." 7.

The same physician is of opinion that "no one who lives long in or near a malarian district, is ever, for a single hour, free from some disease of the digestive organs." The mind, he considers to be enfeebled as well as the body, in these localities, which is evinced by the quiet and unresisting manner in which they succumb to the wretchedness of their lot. "Their dulness and apathy indicate an equal degree of mental as of physical paralysis." The following picture is deplorable.

"In the year 1836," says one of the medical officers of the West Derby Union, "I attended a family of thirteen—twelve of whom had typhus fever, without a bed in the *cellar,* without straw or timber shavings—frequent substitutes. They lay on the floor, and so crowded that I could scarcely pass between them. In another house I attended fourteen patients: there were only two beds in the house. All the patients lay on the boards, and during their illness never had their clothes off. I met with many cases in similar conditions; yet amidst the greatest destitution and want of domestic comfort, *I have never heard, during the course of twelve years' practice, a complaint of inconvenient accommodation.*" 10.

The depressing effects of malaria on mind and body induce to the taking of stimulants and also of opium. Godfrey's Cordial is the chief article exhibited to children. Dr. S. remarks that the fever of the metropolis at present may be called "a new disease"—being totally different from that which he was accustomed to see for a long series of years. "It is as different in its symptoms, and requires as opposite remedies, as any two diseases in the catalogue of nosology." The cause of this change is totally unknown to Dr. Smith. The fact itself is not very encouraging to the theorist—nor, indeed, to the practitioner.

"Of the nature and extent of the change which has taken place in the present instance, I may, in some measure, enable the Commissioners to judge by this circumstance. Formerly there was scarcely a day in which it was not necessary to take blood from some of the patients in the hospital; the inflammatory nature of the disease was the obvious and prevailing one, and blood-letting was indispensable to stop the progress of active inflammation in some vital organ. Now no case presents itself with any indication of active inflammation, and blood-letting is practised in the hospital scarcely four or five times in the year. Formerly wine, brandy, ammonia, anything in the shape of stimulus, was found to aggravate the disease, and was never prescribed excepting in cases attended with unusual prostration, or in the last stage of the malady: now the prostration is from the first so urgent that there is no case which requires any treatment at all that does not stand in need of stimulants; and such remedies at present often save life, whereas before they would probably have destroyed it; and this arises from a total change in the character of the disease." 18.

Our author considers the immediate and direct cause of fever to be a poison generated during the decomposition of animal and vegetable matters. But what can cause such dissimilitude in the fevers of one year as compared with others? The old answer—epidemic constitution—in other words—*ignoramus*. But although we know not what this *tertium quid* is, we know, or think we know, what will cure, or prevent it—sewerage and ventilation.

2. *Mr. N. B. Bagshaw.*—As Dr. Smith's grand remedial and preventive measures were water and air, Mr. Bagshaw's favourite is—SOLAR LIGHT.

"During a practice of 30 years in a densely populated neighbourhood, my attention has been repeatedly drawn to the influence of light, not only as a most efficient means of preventing disease, but likewise as tending materially to render disease milder when it occurs, and more amenable to medical and other treatment. Dupuytren (I think) relates the case of a lady whose maladies had baffled the skill of several eminent practitioners. This lady resided in a dark room (into which the sun never shone), in one of the narrow streets of Paris. After a careful examination, Dupuytren was led to refer her complaints to the absence of light, and recommended her removal to a more cheerful situation. This change was followed by the most beneficial results; all her complaints vanished. Sir James Wylie has given a remarkable instance of the influence of light. He states that the cases of disease on the dark side of an extensive barrack at St. Petersburgh have been uniformly, for many years, in the proportion of three to one to those on the side exposed to strong light. The experiments of Dr. Edwards are conclusive. He has shown that if tadpoles are nourished with proper food, and exposed to the constantly renewed contact of water (so that their beneficial respiration may be maintained), but are entirely deprived of light, their growth continues, but their metamorphosis into the condition of air-breathing animals is arrested, and they remain in the form of large tadpoles. Dr. Edwards also observes that persons who live in caves and cellars, or in very dark and narrow streets, are apt to produce deformed children; and that men who work in mines are liable to disease and deformity beyond what the simple closeness of the air would be likely to produce." 41.

When young people are going to be married, Mr. Ward advises them to choose the largest room they can find, and where the solar light is most freely admitted. Mr. W. thinks that measles are productive of great mortality—and that if plants were grown in the houses of the poor, it would be "laying the axe to the root of the tree."

"That you would do more good to the poor by the adoption of some such plan than can be conceived; that by the introduction of those plants, you would induce the poor to get out into the woods round London, instead of going to the public houses." 44.

3. *Dr. Neil Arnott.*—Dr. A. agrees almost entirely with Mr. Smith.

"Our inquiries gave us the conviction that the immediate and chief cause of many of the diseases which impair the bodily and mental health of the people, and bring a considerable proportion prematurely to the grave, is the poison of *atmospheric impurity* arising from the accumulation in and around their dwellings of the decomposing remnants of the substances used for food and in their arts, and of the impurities given out from their own bodies. The means of removing these sources of injury are—1st, the labour of scavengers for bulky solid matters; 2ndly, the use of sewers or drains, with a sufficient supply of water for liquids and comminuted solids which running water can carry; and 3rdly, modes of ventilation for ærial matters." 50.

4. *Mr. Toynbee* states that, among the poor of the St. George and St. James's Dispensary, nearly all the families have but one room each. He was attending a family where the father, mother, a grown up son in consumption, a daughter of seventeen years, and a child, all slept in the same bed, in a room where the father and four other men worked as tailors during the day! The windows of the poor seldom open at the top, and when opened from below, an inconvenient rush of air takes place, and often does mischief. This prevents proper ventilation of rooms, and the want of ventilation he considers as the main cause of scrofula, which abounds in the district above alluded to. Tailors and other sedentary classes of the poor are very subject to scrofula, and also to gouty affections —the first direct, the second as sequences.

5. *Dr. Guy*—is one of the physicians to the King's College Hospital. Dr. Guy has directed much attention to the diseases of the poor—especially as affected by different avocations and professions.

"1st. Consumption is relatively more frequent in persons working in-doors than in those employed out of doors.

"2nd. In those employed within doors, it is most frequent in men using little exertion.

"3rd. It makes its attack earlier where it is of most frequent occurrence.

"4th. It is very common in the intemperate, and in those exposed to the inhalation of dust.

"5th. It is more frequent in men than women, at least in the metropolis." 89.

The proportion of consumptive cases, in gentlemen, tradesmen, and artisans, were about 16, 28, and 30 per cent. Thus the tradesmen of the metropolis are nearly twice as liable to phthisis as the gentry. This Dr. G. attributes to the confinement of the tradesmen in ill-ventilated shops. We are much inclined to attribute it also to the frequent currents of cold air rushing into close and ill-ventilated apartments.

"In the course of your inquiries have you given any attention to the influence of habits of intemperance on health?——Yes. I have made an exact comparison between the classes most exposed to the temptation of drinking, and those who are not more intemperate than the greater part of the labouring class. I have compared, for instance, the drayman with the labourer, the potboy with the footman, and the licensed victualler with other tradesmen. All these comparisons are very unfavourable to the classes most exposed to the temptation of drinking. I find that, before 35 years of age, more than twice as many draymen die as labourers; and that before the same period the deaths among potboys exceed those of footmen by more than a third.

"You state that men who work out of doors are more addicted to drinking than those who are employed within doors. How do you account for this?—— There are many reasons for it. The man who has much exertion in the open air is not so conscious of the effects of spirituous liquors as the man who leads a sedentary life within doors, nor does his occupation require so much thought or so steady a hand as that of the artisan. Those who work hard, too, think that some kind of fermented liquor is absolutely necessary. Moreover, many labouring men are hired at public-houses, and must recommend themselves by spending their money freely in beer and spirits. The brewers' draymen, the potboys, and the porters at wine-vaults, all have access to intoxicating liquors at all times, and are allowed a large quantity of liquor.

"Does your experience of the poor lead you to believe that habits of intemperance are on the decrease among them ?——Decidedly so. I have taken much pains to ascertain the point, and have no doubt that they are fast improving in this respect, the younger more than the older." 99.

6. *Mr. Liddle.*—This gentleman draws a sad picture of the "White-Chapel Union," to which he is medical officer. The overcrowding of the poor has been much increased by the new improvements that are going on in that neighbourhood. "Some of the larger houses are now converted into lodging-houses which, for the most part, are shamefully crowded."

7. *Dr. Aldis.*—This gentleman is physician to several dispensaries—London Dispensary—Farringdon ditto—Surrey ditto, &c. He finds, generally, one badly-conditioned room occupied by a whole family, badly ventilated and filthy.

"The greatest overcrowding is displayed in Field-lane and its neighbourhood, in the courts leading out of Smithfield, in the courts leading out of Fetter-lane, and the courts and buildings leading out of Fleet-street.

"Some of the worst features of overcrowding are displayed on the Surrey side of the river, in the neighbourhood of the Mint, otherwise the characteristics are common. Within the rooms close offensive smells, the atmosphere quite vitiated, the fæcal smell of the cesspool is often distinguished; the courts are uncleansed and in a dirty condition. Some of the streets in Spitalfields are remarkable for their filth. The most overcrowding in the district of the London Dispensary is detected in the neighbourhood of Artillery-passage, Bell-lane, and Petticoat-lane." 112.

The drains, sewers, and water-supply are in a very neglected condition, and the stench, especially in Summer, most intolerable.

"There is generally a filthy accumulation on the surface of the water in the water-butts. In some courts there is no supply of water; such is the case in Ireland-court and Lusigneas-buildings, Red Lion-street, Spitalfields, which I visited to-day. One woman informed me that her husband lay dead, and that she could not obtain water without the greatest difficulty to wash his 'rags.' I went into her room and found her husband lying dead in a coffin; the room was small, dark, and dirty, and occupied by six children, in addition to the father and mother. Another female represented the place to be 'stinking alive' for the want of water. In the neighbourhood of Field-lane some persons have not even cesspools or privies; all their excrements are thrown into a little back yard, where they are allowed to accumulate for months together; others have a cesspool, but it is not provided with a drain, so that the excrements run into courts or streets, where they remain until a shower of rain washes them into the gutter. These are the places we are called upon most frequently to visit." 113.

The most prevalent diseases were fevers, inflammations, intestinal disorders.

"What is the ordinary physical condition of the population brought up under the physical condition you describe ?——They are emaciated, pale, and thin, and in a low condition, the cases of asthenia being of common occurrence. They complain of sinking, depression of the strength, of spirits, loss of appetite, accompanied by pains in different parts of the body, with disturbed sleep. This feebleness of constitution, among other causes that come before us, may be ascribed to the unwholesome habitations in which they reside. We find, indeed within our experience, considerable differences in respect to this depressed condition. I usually warn pupils that they must not bleed freely in the depressed districts. Bleeding, which may be resorted to freely in rural districts, or in comparatively healthy suburban town districts, cannot be resorted to safely to the same extent in the more crowded and depressed districts. I find, whatever may be their condition as to employment, that more stimulants are requisite in proportion to the depressing influence of the impure atmosphere in which they reside. In all cases of disease, the removal to a purer atmosphere, or from their close rooms to the more spacious and better ventilated wards of the hospital, gives early relief. The depressed and low condition of health in which these people are always found, induces habits of intemperance unfortunately so common among them." 115.

8. *Dr. Rigby*—is senior physician to the Lying-in Hospital, Lambeth. Dr. Rigby has had ample opportunities of witnessing the effects of atmospheric impurities on females pregnant, and on children. The hospital in question, has seldom been free from puerperal fever that often produces frightful ravages that cause the building to be closed occasionally. Although every possible means were employed to ventilate and purify the wards, at these times, the fever generally returned when the hospital was re-opened. It was now found that there were 1500 feet of open ditches near the hospital, receiving the drainage of the poor and dense population of the neighbourhood, and presenting immense exhalations of mephitic gas. This evil was partially relieved at a great expense. The main drain of the building itself was found to be blocked up. All being ultimately cleared away, the fever disappeared.

"In your practice as a physician, have you had occasion to notice in private houses defects such as you experienced and struggled with at the hospital?—I have every reason to believe that in a large majority of cases the ventilation of private houses is very inferior to that of our large hospitals, more particularly as regards the effluvia from drains, &c. The arrangements also for ventilating the sleeping-rooms, especially the servants' bed-rooms, are very defective; the peculiar close, disagreeable smell of these latter chambers must be familiar to all.

"These defects, then, are not confined to the houses of the poor, but exist also in those of the wealthy?——They do; and in houses which have been built many years, to a great extent.

"Can you give some instances?——I may mention one family, the members of which were constantly exposed to the effluvia and stench arising from bad drains; they never had a cook remain with them long without suffering severely in health; as the state of the drains became worse, they all suffered, and at my urgent advice removed to a healthy suburb of London with the most marked effects in the improvement of health. These defects, as regard effluvia and stench arising from defective drainage, exist also among houses comparatively or quite new; for instance, in the Marylebone district, and even among some of the recently built houses of Hyde Park. In the former locality I am at this moment attending a lady in her confinement, whom I have with some difficulty rescued from an attack of puerperal fever, which threatened to assume the malignant form. On being summoned to her when in labour, I was struck by the offensive drain effluvia, which not only pervaded the lower parts of the house, but rose perceptibly from the area as I stood at the hall door, and I cannot help attributing this attack coming on, under all the favourable circumstances of wealth and station, to the deleterious influence to which I have just alluded." 121.

These are the chief results of the examinations of medical men in this over-grown metropolis, where one half of the inhabitants are unaware how the other half live or die! The large provincial towns and cities, as

Liverpool, Birmingham, &c. present the sources of disease in a proportional scale, and almost take away the hope of materially bettering the condition of the working classes, till labour is better paid, and the people become convinced of the imprudence of early marriages, and the dire effects of redundant population!

